# Food Safety in Low and Middle Income Countries

**DOI:** 10.3390/ijerph120910490

**Published:** 2015-08-27

**Authors:** Delia Grace

**Affiliations:** Program Leader Food Safety and Zoonoses, International Livestock Research Institute, P.O. Box 30709, Nairobi 00100, Kenya; E-Mail: d.grace@cgiar.org; Tel.: +254-20-422-3460; Fax: +254-20-422-3001

**Keywords:** food safety, LMICs, equity, health burden

## Abstract

Evidence on foodborne disease (FBD) in low and middle income countries (LMICs) is still limited, but important studies in recent years have broadened our understanding. These suggest that developing country consumers are concerned about FBD; that most of the known burden of FBD disease comes from biological hazards; and, that most FBD is the result of consumption of fresh, perishable foods sold in informal markets. FBD is likely to increase in LMICs as the result of massive increases in the consumption of risky foods (livestock and fish products and produce) and lengthening and broadening value chains. Although intensification of agricultural production is a strong trend, so far agro-industrial production and modern retail have not demonstrated clear advantages in food safety and disease control. There is limited evidence on effective, sustainable and scalable interventions to improve food safety in domestic markets. Training farmers on input use and good practices often benefits those farmers trained, but has not been scalable or sustainable, except where good practices are linked to eligibility for export. Training informal value chain actors who receive business benefits from being trained has been more successful. New technologies, growing public concern and increased emphasis on food system governance can also improve food safety.

## 1. Introduction

Foodborne disease (FBD) is an important issue. The full health effects, as well as the full economic costs, of unsafe food are not known, but the global impact on health, trade, and development is considered enormous: worldwide, hundreds of millions of cases of foodborne disease occur each year costing billions of dollars. This paper reviews FBD in LMICs covering the likely burden of FBD, the importance of FBD to LMICs, the causes of FBD, the most risky foods, the trends in FBD, and management of FBD. The review is a synthesis of a longer report commissioned by the Department for International Development, UK as a learning resource.

## 2. Methodology 

The review was based on a survey of the literature on foodborne disease (FBD) in low and middle income countries (LMICs), discussions with experts working in FBD in LMICs, and several national workshops held as part of FBD research projects led by the International Livestock Research Institute (ILRI) in Africa and Asia. Following the World Bank methodology, low-income economies are defined as those with a GNI per capita, of $1045 or less in 2014; middle-income economies are those with a GNI per capita of more than $1045 but less than $12,736. In this review, FBD is defined as any disease that results from the ingestion of contaminated or naturally hazardous foods; animal source foods (ASF) are defined as foods derived from animals, fish and aquatic animals (including meat, milk, eggs, offal, fish and crustaceans) and produce as fruits and vegetables sold fresh. Many FBD can also be transmitted by other routes such as water or direct contact with infected people or animals, and they are considered foodborne if food plays an important role in disease transmission. Informal or wet markets refer to markets where traditional processing, products and pricing predominate; where many actors do not comply with employment regulations and/or do not pay tax; and, which escape effective health and safety regulation.

## 3. Review Findings

### 3.1. Burden of FBD in LMICs

The full burden of FBD in LMICs is not known but experts believe LMICs bear the brunt of FBD [1,2]. This is plausible given that: high level of hazards are often reported [3]; high prevalence of potentially foodborne pathogens are found in hospital and community surveys of people with diarrhoea [4]; there is a lack of clean water for washing food and utensils (around 750 million people do not have access to clean water [5]; and, use of human sewage or animal waste for horticulture production is common [6].

The structure of the food sector in LMICs compounds the problem. Food systems are heterogeneous and fragmented with large numbers of actors, many small-scale actors, large informal sectors, and relatively little organisation. In China, the food sector is said to consist of “elephants and mice”: that is, sprawling, monopolistic enterprises and tiny household producers [7]. Similarly an expert interviewed for this study, said that, in Africa, “in the retail food sector, there is nothing between the cartel and the corner shop”. While these statements may exaggerate, they do illustrate a fundamental feature of developing country food systems: they are characterised by a great majority of informal sector actors who are difficult to monitor and a few large companies who have incentives to escape or capture regulation.

These structural challenges are compounded by generally poor capacity to enforce regulation in many LMICs. As regards food system regulation, stakeholders cite the following governance challenges: inadequate policy and legislation; multiple organisations with overlapping mandates; out-dated, fragmented or missing legislation; inappropriate standards; lack of harmonisation and alignment of standards; failure to cover the informal sector; limited civil society involvement; and, limited enforcement [8].

### 3.2. Importance of FBD to LMICs

There are few nationally representative surveys of food safety perceptions in LMICs, but smaller studies show high levels of concern over food safety. For example, results from seven countries found food safety was always a concern for consumers and often their single most important concern about food [9]. People’s actions (revealed behaviour) confirm this: when pig diseases were initially reported by the media in Vietnam, the majority of consumers either stopped eating pork, shifted to chicken or went to outlets perceived as safer [10]. Similarly, assessments conducted in the context of Rift Valley fever outbreaks in Kenya found that consumers asked to see butchers’ certificates and demand for ruminant meat dropped as consumers switched to poultry [11]. Food safety has become an issue of enormous public concern in China [12,13]. One survey found Chinese people reported FBD was the second greatest risk they faced in daily life (after earthquakes), and 92% of respondents said they expected to soon become a victim of food poisoning [7].

Food safety can also affect trade. International trade studies have found evidence that the fixed costs of meeting standards tends to favour established exporters and leads to a greater reduction in developing-country exports relative to those in developed countries [14]. FBD can also lead to rejections and high economic costs. For example, the 1991 cholera outbreak in Peru, caused by consumption of water and seafood contaminated by *Vibrio cholerae*, resulted in more than $700 million in lost exports of fish and fish products [15]. More recently, in 2005, malachite green was found in Chinese eels resulting in export losses of at least $860 million [16].

There has been little research on the intersection between gender and food safety in LMICs, but FBD can have important implications for women’s resilience and vulnerability. Firstly, food safety has direct implications for women’s health. Pregnant and lactating women are especially vulnerable to a range of FBD, including listeriosis and toxoplasmosis. (Interestingly, there are many taboos around consumption of food (especially nutritious food), for example, meat is the main target of proscriptions for pregnant women [17]. These taboos tend to protect from some FBD but have the disadvantage of worsening women’s nutrition status.) Secondly, food safety has implications for women’s livelihoods. Women have an important (even dominant) role in many traditional food value chains [18]. Concerns over food safety are often a driver in attempts to modernise and formalise food value chains. However, this may have the unintended consequence of excluding women. For example, although preparing poultry for consumption is traditionally a female role, the modern, private sector, poultry plants in South Africa employ mainly male workers; likewise, while women traditionally dominated milk marketing in West Africa, men predominate in the more recent, peri-urban dairying sector [18]. Lastly, women are risk managers in the realms of food consumption, preparation, processing, selling and, to a lesser extent, production, so gender analysis is important in designing interventions for improving food safety in informal markets.

In many LMICs, most animal source foods and produce are produced by smallholders and sold in informal markets [19,20]. Street food is a large part of the informal sector in most LMICs—the largest in South Africa [21] and therefore a major source of income and employment for the poor, especially women. As concern with food safety increases, and standards ratchet upward, there is a risk that poor producers and value chain actors will be displaced from rapidly growing domestic markets. This has already occurred in export markets where smaller farmers tend to drop out, as they lack the human and financial capital needed to participate in highly demanding markets. For example, in the 2000s both Kenya and Uganda saw major declines (60% and 40% respectively) in small farmers participating in export of fruit and vegetables to Europe under Global Good Agricultural Practices (GAP) [22]. Food safety, therefore, has equity implications, and interventions to improve food safety should not be anti-poor.

### 3.3. Causes of FBD in LMICs

There are many challenges in quantifying the burden of FBD in LMICs. Important diseases may have several transmission routes, and the importance of the food route is not always known. Health information from LMICs is prone to error and under-estimation. Estimates of the disease burden of neglected tropical diseases, several of which are foodborne, have varied considerably over the past decade, and may continue to do so for many years to come [23]. Nonetheless, much progress has been seen in recent years with updated burden assessments from the World Health Organisation (WHO), other burden assessments from the Institute for Health Metrics and Evaluation (IMHE) and an important initiative by the WHO to estimate the global burden of FBD taken forward by the WHO Foodborne Diseases Burden Epidemiology Reference Group (FERG).

Current evidence suggests that foodborne parasites are important causes of disease. (Food has an important role in the transmission of all these parasites and hence they are often called “foodborne parasites”). Some foodborne parasites are very common. For example, worldwide more than one in three people are infected with toxoplasmosis [24] and one in ten with giardiasis [25] (not all these infections are symptomatic). Other foodborne parasites are less common but may have very serious health effects, for example, echinococcosis and cysticercosis. The burden of some of the important foodborne parasites, for which estimates exist, is around 18 million Disability Adjusted Life Years (DALYs) (Table 1), and this is probably an underestimate. Although, detailed breakdowns are lacking, the majority of this burden is probably borne by LMICs, as some foodborne parasites are only found in LMICs, and for other parasites,, the burden is likely to be higher in LMICs because of their higher populations, more challenging conditions, and less access to health services.

**Table 1:** Burden of parasitic diseases commonly transmitted via food.

Parasitic Disease	DALYs	Reference
Cryptosporidiosis	8,372,000	[23]
Amebiasis	2,237,000	[23]
Cystic echinococcosis	1,009,662	[26]
Alveolar echinococcosis	660,434	[27]
Ascariasis	1,310,000	[28]
Trichuriasis (whipworm)	647,400	[28]
Toxoplasmosis	1,200,000	[29]
Foodborne trematodiasis	1,875,000	[30]
Cysticercosis	503,000	[30]

Microbial pathogens are responsible for the majority of the FBD burden in developed countries where they cause 20%–40% of intestinal disease as well as a similar or greater burden due to non-intestinal manifestations of FBD [31,32,33,34,35]. The proportion of diarrhoea due to food in LMICs is not known. Traditionally, most diarrhoea has been attributed to unsafe water (as much as 88% [36]). More recently the 2010 Global Burden of Disease study and a series of reviews of reviews find water, sanitation and hygiene (WASH) risk factors are less important (20%–40% of diarrhoea or less) [37,38,39]. Others question these findings, arguing 60% of diarrhoea is attributable to lack of water, sanitation and hygiene [40] (although much FBD is also attributable to poor hygiene). Even so, as much as 40% of diarrhoea is not accounted for by poor quality water and inadequate sanitation and some of this burden is certainly due to food. The few studies on attribution of intestinal disease from LMICs mainly rely on self-reporting on the cause of disease, which is not very reliable. However, they suggest acute gastro-intestinal disease is common (around one in two people a year) and around one third of cases (12%–55%) are self-attributed to food [41,42,43,44]. The WHO estimates that diarrhoeal diseases have a global burden of 99,727,954 DALYs and that 90% of these are the result of illness in lower income and lower middle-income countries [45]. If we assume conservatively that 10% of the developing country burden of diarrhoea is due to viral and bacterial FBD, this corresponds to around 9 million DALYs each year, (in addition to the burden caused by protozoa). As mentioned, there is reasonable evidence that the burden from non-intestinal health impacts at least as great as the burden from intestinal disease [35,46,47] suggesting at least an additional 9 million DALYs from non-intestinal impacts of FBD in LMICs.

There is a high level of concern among the general public about the presence of chemicals in food. These chemicals include metals, pesticides, growth promoters, chemicals added to food during processing, chemicals added to adulterate food, dioxins and, toxins produced by cooking (polycyclic aromatic hydrocarbons and acrylamides). In LMICs, there is no credible, comprehensive, quantified evidence on the overall impact of chemicals in food on human health [48] but there is solid evidence that some health impacts occur, and there is suspicion that impacts could be substantial. Various studies show: widespread misuse of chemicals; use of obsolete, unduly hazardous and banned chemicals; chemicals present in food above permitted levels; and, foods imported from LMICs having higher levels of chemicals than those imported from developed countries [49,50,51]. The burden of foodborne arsenic has been estimated at an additional 70,000 cases a year of bladder, lung and skin cancer in LMICs [52]. However, the current state of knowledge is not sufficient to estimate the overall health burden associated with chemicals in food in LMICs.

Aflatoxins are naturally occurring, toxic metabolites produced by some species of the *Aspergillus* fungus. They are widespread in crops in tropical and sub-tropical regions, especially maize and groundnuts, and are also found in dairy products and traditionally fermented foods. Ingestion of large amounts of toxin can cause death, and chronic exposure to aflatoxins leads to liver cancer. It is also associated with stunting and immune suppression, but a causal link has not yet been established [53]. Hepatitis B infection appears to worsen the effects of aflatoxins and nearly two thirds of aflatoxin associated liver cancer cases are in hepatitis B positive people [54]. The first global estimate of the health burden of aflatoxins estimated 25,200–155,000 cases of liver cancer resulted from aflatoxin exposure each year [54]. A second study by the same team based on literature from high aflatoxin areas (China and Sub-Saharan Africa) suggested aflatoxins were responsible for 17% of hepato-cellular carcinoma or 88,400 cases (72,800–98,000) equivalent to 1.1 million DALYs [55]. Extrapolating to other regions, this amounts to 127,330 cases and 1.6 million DALYs. Most cases occur in sub-Saharan Africa, Southeast Asia, and China where hepatitis B prevalence is high and aflatoxin exposure in food is common and uncontrolled [54].

Other food safety problems in LMICs cause individual or local tragedies but are not of sufficient scale to be considered global public health priorities. Plants contain some chemicals that are known to be toxic to both animals and humans. Lathyrism, a neurological disease caused by consumption of some types of pulse (*Lathyrus spp.*) has been a problem in south Asia and Ethiopia. Few cases are reported currently, for reasons that are not fully known [56], but probably linked to increasing income, better health care and more diverse diets. Konzo (an acute paralytic illness) and tropical ataxic neuropathy (a chronic illness characterised by sensory deficits) are caused by high dietary cyanogen consumption from insufficiently processed bitter cassava combined, in the case of konzo, with a protein-deficient diet. Around 2000 cases of konzo have been reported over the last 20 years but unofficial reports suggest there may be tens of thousands of unreported cases [57]. 

Seafood poisoning often results from marine toxins. Ciguatoxins in contaminated tropical reef fish cause ciguatera poisoning, the most common marine toxin disease with 10,000 to 50,000 cases a year worldwide (but the true incidence is difficult to ascertain due to under-reporting [58]). Paralytic shellfish poisoning caused by the algae producing the toxin "red tides” is most common in colder waters. Diarrhetic shellfish poisoning is common in Europe but found worldwide and was recently reported in China [59]. Scombrotoxic fish poisoning is a syndrome resembling an allergic reaction caused by eating fish with high levels of histamine produced by bacterial spoilage. It is most associated with consumption of scombroid fish (e.g., tuna, mackerel). Several other marine toxins are known to cause illness but the health burden in LMICs is not known.

Illicit or unrecorded alcohol constitutes about 30% of all alcohol consumed globally, with poorer countries consuming a higher proportion of illicit alcohol [60]. Illicit alcohol may contain methanol either due to incorrectly managed distillation processes, or, more commonly, when methanol is deliberately added to fortify drinks. As reported in the published literature account, methanol poisoning is responsible for fewer than 1000 deaths in any given year. In India, around 2000 deaths have occurred since independence [61].

Food adulteration to increase profits has led to several high profile outbreaks of food poisoning. In Cambodia, in 1996 and 1998, 70 deaths were linked to the drinking of rice wine mixed with pesticides to make it stronger and problems with Sudan Red, melamine in formula milk and malachite have been reported from China [14]. There are also occasional reports of malicious poisonings (e.g., in China, 38 school children died by rat poison when a baker contaminated products of a rival seller to obtain commercial advantage [62]. Accidental contamination may be more common in LMICs as a result of lack of stringent health and safety protocols and these incidents are occasionally reported in the media. For example, in India in 2013, at least 22 schoolchildren died after eating a free school lunch contaminated with insecticide [63]. 

### 3.4. Foods Associated with FBD in LMICs

In countries where good data exist, most FBD results from consuming fresh animal source foods (ASF) (*i.e*., meat, milk, eggs, and aquatic animals) and produce (*i.e*., fresh fruits and vegetables). In LMICs, less fresh food (ASF and produce) is eaten, but the fresh food eaten is more contaminated [3,64,65]. Some representative findings on hazards in fresh foods from a range of ILRI studies on food in informal markets are: only 6% of pork sampled in Nagaland, India complied with standards [66]; only 2% of meat samples in Nigeria complied with standards [67]; 0% of milk samples in Assam complied with standards [68]. Fresh ASF and produce are also the foods most often implicated as causes of FBD cases. Table 2 shows the foods implicated in FBD from three large, comprehensive national studies in the UK, USA and Netherlands one small study in India and one medium-sized, self-reported study from China. In all of these studies, animal source foods and produce are the most common causes of FBD illnesses. In studies which also assessed health burden, the role of animal source food was even higher as the illnesses caused tended to be more serious: for example ASF were responsible for 82% of DALYs in the Netherlands and 51% of deaths in the USA. 

**Table 2:** Types of food responsible for foodborne illness in some countries.

Countries	Animal Source Food %	Produce (Fruit & Vegetables) %	Other %	Survey	Reference
UK	78	10	11	National	[32]
Netherlands	63	7	30	National	[34]
USA	48	46	6	National	[69]
India	71	29	0	One city	[70]
China	32	40	28	Province	[44]

The parasitic and microbial pathogens responsible for most of the health burden are often acquired from animal source food and meat consumption appears to be a strong predictor of FBD mortality. A cross-country study found that for every additional metric tonne of meat consumed per 100 people, food borne disease mortality increased by 6% [71]. Pesticides are mostly transmitted through vegetables and fruit but are also commonplace in animal source foods in LMICs [72,73,74].

### 3.5. Trends in FBD in LMICs

Despite considerable investments in food safety, the EU and USA have seen no change or a deterioration in the number of cases of most (but not all) foodborne diseases over the last five (EU) or ten (USA) years [75,76]. (A notable exception is salmonellosis in Europe, which is declining, largely due to vigorous control in poultry [77]). A review argues that the investments in food safety over the last 20 years have had limited impact, not because the strategies are ineffective, but because of other factors such as globalisation, changes in eating habits and changes in farming practice increasing risk [78]. Because there is no accurate reporting of foodborne disease in LMICs it is more difficult to monitor trends. In the USA and Europe, food safety was an issue of intense concern during the periods of most rapid industrialization and urbanization [79,80], and this concern is now evident in more rapidly industrialising LMICs. Many LMICs are undergoing rapid agricultural intensification, which may increase the risk of FBD [81]. A review of agricultural intensification and human health in the Mekong found links between irrigation and fish borne parasites; livestock manure and contaminated produce; antimicrobial use and transfer of resistant bacteria through food; and, pesticide use and contaminated foods [82]. Another a multi-country review found that a 1% increase in crop output per hectare was associated with a 1.8% increase in pesticide use and that pesticides were more weakly regulated in countries undergoing intensification, implying greater risk of food contamination [83]. 

Diarrhoeal illness is decreasing overall in LMICs. Notwithstanding, it is possible that the share of FBD is static or increasing, especially given the good success in improving access to potable water. (Between 1990 and 2012, over 2.3 billion people gained access to improved drinking water, which now reaches 89% of the world’s population [5]). However, the IMHE estimates that there have been large declines in diseases that are mainly transmitted by food (e.g., non cholera salmonellosis and campylobacteriosis) [30]. Given on the one hand, no obvious decline in foodborne disease in countries with good records, and on the other hand, an estimated global decline in some diseases mainly transmitted by food, it is difficult to draw over-arching conclusions about trends in foodborne disease.

Indeed there are some reasons why FBD may increase in some LMICs. Firstly, consumption of fresh, perishable, more risky foods is growing rapidly, driven by increasing population, income, urbanization and globalization. Even in East and South Africa, which are not the fastest growing markets, per capita expenditure on perishables is set to quadruple by 2040 and the total market size increase by a factor of eight [84]. Secondly, in response to increased demand, food chains are becoming longer and more complex increasing the spread of hazards. Mature and well-governed value chains may be able to reduce FBD by insisting on high standards along the value chain and conducting testing. However, in LMICs the expansion of value chains is happening in advance of effective governance, increasing risk. In China and Vietnam, for example, changing industry structure, rapid market development, rapidly changing prices of products and inputs, low profit margins, lack of bargaining power in key players and lack of government support to stabilise markets all put high pressure on value chain actors to cut corners and sacrifice food safety [85]. This is driven by the dynamics of agricultural investment: high prices drive a rush of investment that raises production leading to lowered prices and waning investment which persists until supply is so low that prices spike. The end result is high levels of volatility that chill investment in food safety [86].

Climate change is on-going and can increase foodborne disease by bringing novel vectors and pathogens into temperate regions or by temperature-associated changes in contamination levels. A recent extensive literature review concluded that campylobacteriosis and salmonellosis were most likely to increase with air temperature; campylobacteriosis and non-cholera vibrio infections with water temperature; cryptosporidiosis followed by campylobacteriosis with increased frequency with precipitation; and cryptosporidiosis followed by non-cholera vibrio in association with precipitation events. Listeriosis was not associated with temperature thresholds, extreme precipitation events, or temperature limits [87].

For other trends it is not clear whether the net impacts on FBD will be positive or negative. Supermarkets are rapidly increasing in LMICs. In Mexico, Central America and Southeast Asia their share is 10%–50% of the retail market while in sub Saharan Africa (outside South Africa) and south Asia the share is less than 10%. Moreover, in India, supermarkets would have to grow at rates of 20%, for 20 years to reach just 20% of market share [88] suggesting the near term focus should be on informal markets. It is commonly believed that supermarket food is safer than informal market food, however, in the case of milk in Assam, Kenya, and Tanzania as well as meat in Vietnam and Kenya the food sold in the formal sector was no better (and sometimes worse) at meeting standards than food sold in the informal sector [89].

Alongside the ‘fresh food revolutions’, there is also growing demand for processed, convenience and snack food. Experts consulted in our study, suggested repeated scares over fresh food safety are prompting switches to packaged and processed food. Processed food is less likely to contain biological hazards, but may be more likely to contain chemical hazards. For example, in China, recent years have seen use of melamine to increase the apparent protein level of baby milk; ink to colour noodles; and sodium borate used to make cheap pork resemble beef [80]. A meta-review of studies of acute food poisoning sourced from Chinese academic databases for the period 2000–2010, covering 2,387 individual incidents of acute foodborne illnesses found food additives were responsible for 9.9% of incidents, 3.5% of illnesses and 11.6% of deaths [90].

Many experts believe the emerging markets will eventually converge with the richer countries, where food is generally safe (with some tragic exceptions) [91]. Indeed, panic over food safety can be a driver for improvement. From this perspective, the situation in China where a widely publicised finding is that half establishments are failing food inspection may be more positive than the situation in India where no reports on food safety inspection or results are publically available [13].

### 3.6. Managing FBD in LMICs

The limited literature on domestic food safety regulation in LMICs shows that we do not yet have good examples of standards and approaches that can address food safety where risks are pervasive, costs of compliance are high, and enforcement capacity is weak [92]. For some interventions, there is little evidence for benefit or sustainability. Nonetheless, other initiatives show promise, and a smaller number have been able to demonstrate sustained and scalable benefits.

It is commonly believed that many problems of food safety and disease can be ameliorated by transiting to “modern” agro-food systems. For example, in China, wet markets have been singled out as major sources of poultry disease and there have been several attempts to ban them [93]; while in Vietnam the plan for modernisation of agriculture aims to encourage large-scale intensive farms and reduce smallholder production. Many also believe that intensive, agro-industry is required to meet food demand. For example, the FAO reports “*As it stands, there are no technically or economically viable alternatives to intensive production for providing the bulk of the livestock food supply for growing cities*” [94]. However, when the informal sector offers better prices to both farmers and consumers, as is typically the case, the formal sector is uncompetitive leading to underutilised capacity [95]. In addition, the intensive sector has been associated with emergence of new human diseases including swine influenza in Mexico, salmonellosis in the 1980s (caused by *Salmonella enteritidis* phage type 4), and “mad cow disease” in the UK [79]. The emergence of Nipah virus in Malaysia was primarily driven by intensification of the pig industry combined with fruit production in an area already populated by infected fruit bats [96,97]. There are also many concerns about the environmental externalities of intensive agriculture including land and water pollution and deforestation [81,82]. Given these findings, caution is advised in assuming modern agro-industry and retail will be a solution for FBD in LMICs.

An important principle of food safety management is that risks must be managed along the “farm to fork” pathway and that some risks are most effectively managed on farm. Several initiatives, with safer foods at least one of their goals, have focused on farmer training. This typically covers hygiene, and safe use of chemicals and drugs, often taught as part of “good agricultural practices” (GAP). There have been many reports on small scale or pilot training of farmers and these often show improvements in practices and hygiene [98,99]. However, there is less evidence for success at large scale. A recent systematic review of Farmer Field Schools, which had a strong emphasis on integrated pest management and reducing pesticides, showed that farmers in the programme had higher yields and used less pesticide, but there was no evidence to show that benefits were either sustained or scalable [100]. While some small farmers have been able to comply with GAP required for exporting their goods, there is less information on domestic GAP programmes and the limited literature suggests impact seems is low [101,102].

Initiatives aimed at training informal value chain actors seem to have had higher success [103,104,105]. However, the only meta-analysis of interventions to train food handlers, found trained handlers had around 30% improvement in knowledge over controls (n = 9 studies) and 70% improvement in practices, but this was based on self-reported practices, which are prone to exaggeration [106]. In Kenya and Assam, initiatives to train milk traders and provide an enabling environment were effective, economically attractive, scalable and sustainable. Currently an estimated 6.5 million consumers are benefiting from safer milk sold by trained and certified traders in the two countries [107,108].

Where value chain actors are not using food safety technologies, simple innovations such as food grade containers or chlorinated water can result in substantial improvements to food safety and quality. Other technologies are effective and affordable but are not commonly used: for example, adding lactoperoxidase to preserve milk; using chlorine washes to reduce bacteria on carcasses; and, using mycotoxin binders to reduce aflatoxins in animal feeds. In several of these cases, technologies are not used in Europe or other developed countries because of secondary considerations which may be relevant to rich countries but are less so to poor countries.

There is general consensus that most developing country governments are not able to ensure the safety of most food consumed in domestic markets. There has been interest in restructuring food safety governance. A single unified structure or an integrated system is likely to be more effective, but when it is not possible because of historical or political reasons a national food control strategy can identify roles [9]. More rational food safety governance systems is important, but experience has shown that even when policies and regulations are good, they are rarely translated into implementation

## 4. Conclusions 

There is reasonable evidence that LMICs bear the brunt of FBD; that developing country consumers are concerned about FBD; that most of the known burden of FBD disease comes from biological hazards; and, that most FBD is the result of consumption of fresh, perishable foods sold in informal markets. While we don’t have good data on the burden of FBD in LMICs, microbial pathogens may cause a burden of 18 million DALYs a year, foodborne parasites at least the same, and aflatoxins 1–2 million DALYs. The full burden of chemical hazards is not known.

Food safety has been neglected in LMICs, where most efforts to reduce diarrhoea have focused on water, sanitation and hygiene. However, these interventions and improvements still leave a large proportion of diarrhoeal disease un-managed and evidence is growing that FBD may be an important contributor to health burdens. Moreover, as good progress continues to be made in attaining clean water and sanitation goals, FBD may become more salient. FBD has been increasing in some developed countries and is likely to increase in LMICs as the result of massive increases in the consumption of risky foods (livestock and fish products and produce) and of lengthening and broadening value chains, bulking more food and increasing the distance between production and consumption. Rapid livestock and fish intensification may also lead to increased FBD as may the growing urban and peri-urban vegetable production relying on wastewater and untreated human and/or animal waste. 

There is limited evidence on effective, sustainable and scalable food safety interventions but some promising approaches. Building on the existing food system may be more successful than attempting to impose completely new systems. Given the importance of FBD, better impact assessment of interventions to improve food safety is a priority. There are opportunities to improve food safety through technologies, value chain innovations and restructuring of food safety governance, but the feasibility and effectiveness of these is not well understood. However, the widespread concern over food safety in LMICs and the growing evidence of the associated health burden and economic costs, make it likely that this area will receive greater attention in future.
